# Involvement of Thalamus in Initiation of Epileptic Seizures Induced by Pilocarpine in Mice

**DOI:** 10.1155/2014/675128

**Published:** 2014-03-20

**Authors:** Yong-Hua Li, Jia-Jia Li, Qin-Chi Lu, Hai-Qing Gong, Pei-Ji Liang, Pu-Ming Zhang

**Affiliations:** ^1^School of Biomedical Engineering, Shanghai Jiao Tong University, 800 Dong-Chuan Road, Shanghai 200240, China; ^2^Department of Neurology, Ren Ji Hospital, School of Medicine, Shanghai Jiao Tong University, Shanghai 200127, China

## Abstract

Studies have suggested that thalamus is involved in temporal lobe epilepsy, but the role of thalamus is still unclear. We obtained local filed potentials (LFPs) and single-unit activities from CA1 of hippocampus and parafascicular nucleus of thalamus during the development of epileptic seizures induced by pilocarpine in mice. Two measures, redundancy and directionality index, were used to analyze the electrophysiological characters of neuronal activities and the information flow between thalamus and hippocampus. We found that LFPs became more regular during the seizure in both hippocampus and thalamus, and in some cases LFPs showed a transient disorder at seizure onset. The variation tendency of the peak values of cross-correlation function between neurons matched the variation tendency of the redundancy of LFPs. The information tended to flow from thalamus to hippocampus during seizure initiation period no matter what the information flow direction was before the seizure. In some cases the information flow was symmetrically bidirectional, but none was found in which the information flowed from hippocampus to thalamus during the seizure initiation period. In addition, inactivation of thalamus by tetrodotoxin (TTX) resulted in a suppression of seizures. These results suggest that thalamus may play an important role in the initiation of epileptic seizures.

## 1. Introduction

Temporal lobe epilepsy (TLE) has been associated with hippocampal sclerosis and pathological changes in the closed neighboring structures, including entorhinal cortex, amygdala and dentate gyrus [1–3]. Scalp electroencephalogram (EEG) recordings from patients with TLE usually demonstrate interictal and ictal epileptiform abnormalities over the mid/anterior temporal region [4]. Studies using depth electrodes further confirmed the electrographic origin of these seizures in the hippocampal formation [5]. However, recent studies have shown that the pathological substrate of TLE is not confined to the temporal lobe. Structural and metabolic imaging studies, such as magnetic resonance imaging (MRI), functional MRI, diffusion tensor imaging, and positron emission tomography, demonstrated abnormalities extending from hippocampus to subcortical structures and bilateral cortical regions in the precentral gyrus in patients with TLE [6, 7]. In clinical practice, resection of epileptic focus within the temporal lobe might still fail to control the seizures in some patients with TLE. The main factor contributing to these failures is probably the incomplete resection of the epileptogenic zone or that the patients might suffer from a complex epileptogenic network, which may involve the subcortical structures [8, 9]. In brief, more and more studies have shown that the hippocampal formation, although very important, may be not the only epileptogenic zone of TLE. So we should extend investigations outside the temporal lobe and focus on the neuronal circuits that may support the seizures [10, 11].

Thalamus is one of the potential critical components in the neuronal circuits that may take part in the initiation and spread of seizures in TLE. It has rich and reciprocal connections with cerebral cortex and limbic system [12, 13]. These connections participate in relaying sensory and motor signals, along with regulation of consciousness, sleep, and alertness in the normal physiological condition and also could be involved in the genesis or propagation or both of focal and generalized seizures in the pathological state [14].

The role of thalamus has been well demonstrated in animal models of absence seizures which are the most pure form of generalized epilepsy [15]. The epileptic network was defined as a reciprocal circuit involving the neocortex and thalamic relay nuclei, and the thalamic reticular nucleus served as a key modulator. In this network, the cortex provided the excitatory drive, and the thalamic relay nuclei organized the drive into the epileptic spike wave pattern [16].

However, the role of thalamus in TLE is still less understood. Recently, some clinical trials targeted various thalamic nuclei as therapeutic electrical stimulation zones in attempt to control the intractable epilepsy, mostly TLE, with varying degrees of seizure reduction in more than half of the patients [17]. Moreover, there were evidences of thalamic involvement in seizures of TLE in patients, although the role of the thalamus was not well defined [9]. In animal models of TLE, studies have provided the histopathologic evidence for cell loss in the medial subdivisions of thalamus that coupled with synaptic alterations, which could enhance the excitability of thalamic seizure circuits [18]. Enhancement of GABA activity in the medial dorsal nucleus (MD) of thalamus resulted in a significant reduction of seizure duration [18, 19]. Further investigations proposed that thalamus could act as an excitatory amplifier through divergent-convergent circuits in the seizures [10, 20]. Besides MD, other nuclei of thalamus may also participate in seizures. Electrical stimulation of the reticular nucleus suppressed the limbic motor seizures in a hippocampal kindling model [21]. Electrical stimulation of the anterior nucleus (ANT) showed an improvement in seizure control in the status epilepticus (SE) model of TLE [22], and the possible mechanism was that the stimulation of ANT caused decreases in concentrations of glutamate and increases in GABA in hippocampus [23]. In the kainic acid model of TLE, high-frequency electrical stimulation of the parafascicular nucleus (PF) interrupted the ongoing hippocampal paroxysmal discharges, and the administration of NMDA antagonist and GABA_A_ agonist suppressed the hippocampal discharges, whereas NMDA agonist and GABA_A_ antagonist increased the hippocampal discharges [24]. These findings indicated that several nuclei of thalamus had close relation with the hippocampus in the neuronal circuits of seizures. However, in the study of absence seizures, enhancement of glutamate activity of PF significantly suppressed the spike-and-wave discharges [25]. It is interesting that activating PF had the opposite effects on epileptic discharges in the temporal lobe epileptic seizures and the absence seizures, calling for more investigations on the role of PF in seizures.

Studies mentioned above mainly investigated the role of thalamus by pharmaceutically modulating or electrical stimulating the thalamus nuclei. In the present study we investigated the role of thalamus in the development of seizures by computational methods. We used multichannel microelectrode techniques to obtain the local field potentials (LFPs) and single-unit discharges from CA1 of hippocampus and PF of thalamus during the development of epileptic seizures induced by pilocarpine in mice. Computational methods from symbolic dynamics and information theory were used to analyze the electrophysiological characters of neuronal activities and the information flow between thalamus and hippocampus. We found that LFPs became more regular during the seizure in both hippocampus and thalamus, and in some cases LFPs showed a transient disorder state at the seizure onset. The information tended to flow from thalamus to hippocampus during seizure initiation period, and inactivation of thalamus by tetrodotoxin (TTX) resulted in a suppression of seizures. These findings indicated that thalamus may play an important role in the initiation of epileptic seizures.

## 2. Materials and Methods

### 2.1. Animals

Experiments were performed on 4- to 6-month-old male C57BL/6 mice. The mice were housed in individual cages with food and water* ad libitum*, and kept in a 12 h light/dark cycle. All animal experimentations were approved by the Ethic Committee, School of Biomedical Engineering, Shanghai Jiao Tong University. All efforts were made to minimize the animal suffering and reduce the number of animals used in the experiments.

### 2.2. Recording Electrodes

**Figure 1:**
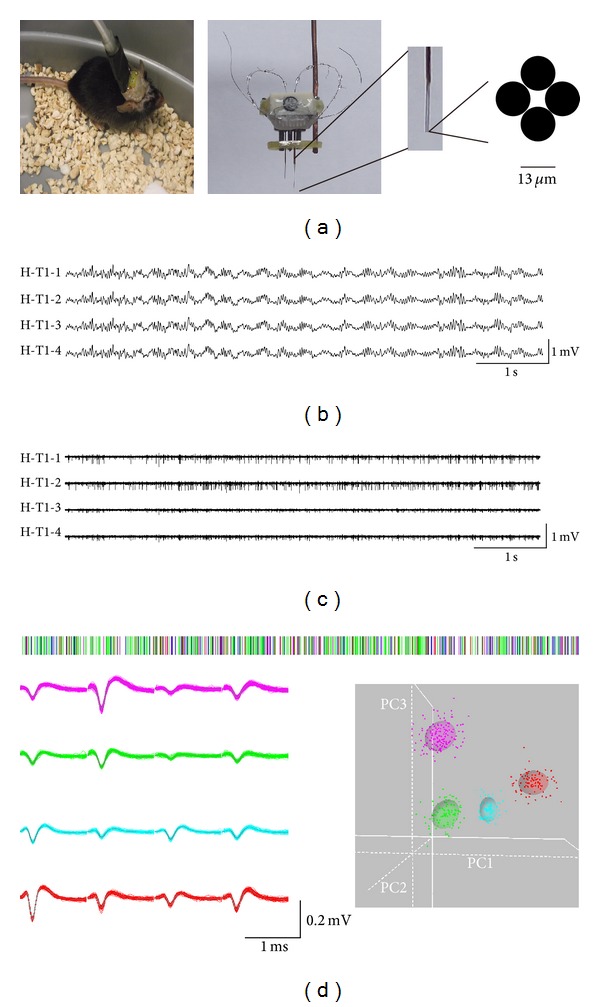
Multichannel microelectrode recordings. Two bundles of electrodes were inserted into the CA1 region of the left hippocampus and PF region of the left thalamus, respectively. Each bundle consists of two tetrodes and each tetrode is formed of four insulated nickel-chrome alloy electrodes (diameter of 13 *μ*m, impedance of 0.5–1.0 MΩ). For each electrode, we extracted single-unit activities and LFPs. Single-unit activities were obtained by filtering the recording at 300–6000 Hz and sorting the filtered data using OfflineSorter software (Plexon Co, USA). To obtain the LFPs, we filtered the data at 0.5–100 Hz. (a) Multichannel microelectrode. The enlarged view shows one bundle of electrodes consisted of two tetrodes and the cross-section through a tetrode. (b) LFPs from a tetrode in the normal hippocampus. (c) Multiunit activity (MUA) of the same tetrode. (d) Example of single-unit activity. The upper trace is the spike train sorted from the MUA in (c). The spikes were from four neurons denoted by four colors. The left bottom traces show the superimposed single waveforms of spikes of four neurons. The right bottom figure shows the four clusters in the feature space, which were projected by the spikes of the four neurons, by using the principal component analysis (PCA) method. H, hippocampus; T, thalamus; T1&T2, Tetrode 1 and Tetrode 2 in a bundle. For example, H-T1-2 means the second channel of Tetrode 1 in hippocampus.

The 16-channel electrodes, consisting of two independently movable bundles of 2 tetrodes, were secured to a recording microdrive (Figure 1(a)). The microdrive was constructed as described [26]. Each tetrode was formed of four twisted polyester insulated nickel-chrome alloy wires (diameter, 13 *μ*m; STABLOHM 675, California Fine Wire Co, USA) with an impedance of 0.5–1 MΩ.

### 2.3. Surgery

Mice were handled for about one week prior to surgery to minimize the potential stress of human interaction. During the surgery, mice were anesthetized by pentobarbital sodium (100 mg/kg) intraperitoneally and mounted in a stereotaxic frame (51600, Stoelting Co, USA). The skin covering the skull was opened and the skull was exposed. The skull was perforated using a high speed dental drill (K.1070 High Speed Rotary Micromotor Kit, Foredom Co, USA) with 1.2 mm diameter drill tips. Seven small holes were drilled: five for the positioning of anchor screws and two for the placement of electrodes. Two cortical screws placed in the bilateral frontal bone were used as the reference and ground. Two bundles of electrodes were implanted into the target regions. The recording microdrive was then fixed to the skull using zinc phosphate cement (Hoffmann Dental Manufaktur GmbH, Germany). Throughout the experiments, body temperature was maintained at 37.5°C using a closed-loop animal blanket system (SS20-2, Huaibei Zhenghua Biologic Apparatus Facilities LTD Co, China). Animals were allowed at least one week for recovery before seizure induction.

### 2.4. Seizure Induction

Mice were injected intraperitoneally with atropine sulfate (1 mg/kg) and 30 minutes later with a single dose of pilocarpine (300 mg/kg). Their behavior was scored according to the Racine scale [27], and status epilepticus (SE) was defined as continuous Stage 3 or more serious seizures. SE was terminated after 1 h by injection of diazepam (10 mg/kg).

### 2.5. Electrophysiological Recordings

A bundle of electrodes was inserted in CA1 of the left hippocampus (with bregma as the reference, anteroposterior (AP), −2.3 mm; mediolateral (ML), −2.1 mm; dorsoventral (DV), −1.0 to −1.4 mm) [28]. The other bundle of electrodes was implanted into the left PF (AP, −2.3 mm; ML, −0.5 mm; DV, −3.0 to −3.4 mm). Each bundle of electrodes can be independently regulated in the depth direction. The depth of the CA1 region was determined electrophysiologically. The initial depth was 1.0 mm below the dura. Then the electrodes were lowered slowly. When the electrode reached the pyramidal cell layer of the CA1 region, single-unit activities were recorded. Similarly, the electrodes in the PF region were adjusted between the depth of 3.0 mm and 3.4 mm until single-unit activities were recorded. The electrodes were lowered to the target areas at least one day before the seizure induction.

The data were amplified (×500), filtered (0.5–6,000 Hz), and stored in a computer (16 bits AD converter, 40 kHz sampling rate) using OmniPlex D Neural Data Acquisition System (Plexon Co, USA).

### 2.6. Histological Experiments

At the end of the electrophysiological recordings, mice were deeply anesthetized with pentobarbital sodium (120 mg/kg) and perfused transcardially with 20 mL of 0.9% saline solution followed by 50 mL of fixative (4% paraformaldehyde in 0.1 M sodium phosphate buffer (PBS), pH 7.4). The brains were then removed and postfixed in 4% paraformaldehyde 0.1 M PBS solution at 4°C for 24 h, equilibrated in 30% sucrose in 0.1 M PBS at least overnight. The whole brain was frozen and sectioned coronally on a cryostat microtome set at 70 *μ*m. Electrodes outside the target regions were excluded in the analysis.

### 2.7. Data Analysis

The data were replayed and filtered with two different band-pass filters: 0.5–100 Hz to obtain LFPs and 300–6,000 Hz to obtain spikes as illustrated in Figures 1(b) and 1(c), respectively. The spike data were later sorted into single-unit activities using the Offline Sorter software (Plexon Co, USA). Single-unit activities were sorted according to a threshold and shape detector using principal component analysis method (Figure 1(d)). LFP signals were resampled by the frequency of 500 Hz. In addition, LFPs recorded from 4 channels of a tetrode were highly similar since the distance between wires in a tetrode was only about 15–25 *μ*m. Therefore we used the mean of LFP signals recorded from the four channels in a tetrode to represent the LFP signal of the tetrode. The term of LFP below refers to the mean value.

#### 2.7.1. Cross-Correlation of Single-Unit Activities

We analyzed the synchronization of firing activities between the recorded neurons by calculating the cross-correlation function (CCF). CCF is one of the most commonly used methods to evaluate the synchronization of firing activities between neurons. In order to calculate CCF, each neuron's spike train is symbolized into “0” and “1” within a time bin (time bin = 2 ms, to ensure there is only up to one spike in one time bin), where “1” means that neuron *i* fires in the *t*th time bin and “0” means no firing. CCF is calculated as

(1) $$\begin{array}{c} \text{CCF}\left(\Delta t\right)=\frac{N\sum_{t=1+\left|\Delta t\right|}^{N-|\Delta t|}r_{1}\left(t\right)r_{2}\left(t+\Delta t\right)}{\left(N-2\left|\Delta t\right|\right)\sqrt{\sum_{t=1}^{N}r_{1}{\left(t\right)}^{2}\sum_{t=1}^{N}r_{2}{\left(t\right)}^{2}}}, \end{array}$$

where *r*_*i*_(*t*) for *i* = 1, 2, denotes the spike train generated by the *i*th neuron at the moment *t* and *N* indicates the length of spike train [29]. The maximum of CCF reflects a maximal synchronization of two sequences when the *r*_1_(*t*) was postponed for lag Δ*t*. In order to examine the dynamics of neuron population activities, we used a moving window with length of 5 seconds and shifted in 1-second steps to calculate CCF. In each window,  Δ*t* ranges from −200 ms to 200 ms.

#### 2.7.2. Redundancy of LFP

Various methods have been used to analyze the temporal evolution of brain activities from EEG or LFP recordings, ranging from traditional linear methods to nonlinear methods [30]. To some extent, the nonlinear methods are superior to the traditional linear methods in extracting information from EEG or LFP data [31]. However, these nonlinear methods assume that the signal is stationary and originates from a low dimensional nonlinear system. Without doubt, EEG or LFP is nonstationary signal. Recently, Bandt and Pompe proposed an ordinal time series analysis method, that is, permutation entropy, which measures the irregularity of nonstationary time series [32]. This method concentrates on the order relations between the values of a time series but not the values themselves. The advantages of this method are its simplicity and low complexity in computation without further model assumptions [32, 33]. Furthermore, the Bandt-Pompe method is robust in the presence of observational and dynamical noise [34]. Over the last few years, the permutation entropy and related metrics have emerged as particularly appropriate complexity measures in the study of time series from biological systems, such as the brain or the heart. It is mainly used in researches on epilepsy, anesthesiology, and cognitive neuroscience [33].

Redundancy is equal to one minus normalized permutation entropy; thus, it is also an index to measures the irregularity of nonstationary time series, with the opposite trend to the permutation entropy. Here, the redundancy of LFPs was analyzed to deduce the signals' irregularity changing during the development of epileptic seizures.

Given a time series of length *L*, {*x*_1_, *x*_2_,…, *x*_*L*_}, a vector is generated by an embedding procedure: *S*_*t*_ = [*x*_*t*_, *x*_*t*+*τ*_,…, *x*_*t*+(*m*−1)*τ*_], where *m* and *τ* are the embedding dimension and the lag, respectively. *S*_*t*_ is rearranged in an ascending order, *x*_*t*+(*j*_1_−1)*τ*_ ≤ *x*_*t*+(*j*_2_−1)*τ*_ ≤ ⋯≤*x*_*t*+(*j*_*m*_−1)*τ*_. We set *j*_*r*−1_ < *j*_*r*_ in the case of *x*_*t*+(*j*_*r*−1_−1)*τ*_ = *x*_*t*+(*j*_*r*_−1)*τ*_. For *m* different numbers, there are *N* = *m*! = (1 × 2 × ⋯×*m*) possible ordinal patterns *π*_*i*_, *i* = 1,…, *N*, also called permutations. Then we count the occurrences of the ordinal pattern *π*_*i*_, which is denoted as *C*(*π*_*i*_), and the relative frequency is calculated by *p*(*π*_*i*_) = *C*(*π*_*i*_)/[*L* − (*m* − 1)*τ*], *i* = 1,2,…, *N* [35].

Using the empirical probabilities *p*(*π*_*i*_), we compute the permutation entropy [32] of the time series, which is defined as

(2) $$\begin{array}{c} H=-\overset{N}{\underset{i=1}{\sum }}p\left(\pi_{i}\right)\cdot \text{lo}{\text{g}}_{2}p\left(\pi_{i}\right). \end{array}$$

The index *i* runs over all possible ordinal patterns. *H*_max⁡_ = log_2_ *N*. If *H* is divided by *H*_max⁡_, one obtains the normalized permutation entropy, which takes values from 0 to 1.

Here we used a moving window of length 2,500 sample points corresponding to 5 s duration, which was shifted forward in 1 s steps. Bandt and Pompe recommended that the length of the ordinal patterns should be set as *m* = 3,…, 7 [32]. When *m* was set as 3, 4, or 5, the results were similar in our analysis (data not shown). For longer patterns, the duration of the moving window would have to be increased in order to reliably estimate these relative frequencies, and then the temporal resolution would become too low to track the dynamics of electrical activities during the epileptic seizures. So *m* was set to 3, yielding 3! = 6 possible different patterns. The lag *τ* = 1. Each moving window contains 2,500 −3 + 1 = 2498 patterns of length *m* = 3 and thus is large enough to estimate the relative frequencies of single patterns.

The redundancy is defined as follows [36]:

(3) $$\begin{array}{c} R=1-\frac{-\sum_{i=1}^{N}p\left(\pi_{i}\right)\cdot \text{lo}{\text{g}}_{2}p\left(\pi_{i}\right)}{\text{lo}{\text{g}}_{2}N}=1-\frac{H}{H_{\max}}. \end{array}$$

The redundancy (*R*) takes values in the range [0, 1]. When only a single ordinal pattern occurs, *R* reaches its maximal value of one. If all ordinal patterns occur with equal probability, *R* becomes zero. In other words, “monomorphic” LFP signals map to high redundant ordinal time series consisting of very few ordinal pattern, while “pleomorphic” LFP signals will map to less redundant ordinal time series consisting of many ordinal patterns [36].

#### 2.7.3. Directionality Index between Hippocampus and Thalamus

Various methods have been proposed to analyze the coupling direction between neuronal signals from different brain areas, such as Granger causality method [37] and state-pace and phase-dynamic approaches [38]. The Granger causality methods can be successfully applied to linear models but cannot be directly applied to nonlinear time series. The state-space approach requires optimal embedding parameters and the phase-dynamics approach requires strong oscillations in signals. The methods based on information theory are also proposed to estimate the coupling direction between neuronal signals, including transfer entropy [39] and conditional mutual information [40]. Recently, the permutation analysis and conditional mutual information were integrated, which was called permutation conditional mutual information (PCMI), to estimate the coupling direction between neuronal signals from different neuronal populations [41]. The stimulation results show that this method is superior to the conditional mutual information method and the Granger causality method for identifying the coupling direction between unidirectional or bidirectional neuronal populations [41].

For these reasons above, PCMI between LFPs recorded from hippocampus and thalamus was computed to assess the coupling direction between the two brain areas.

The LFPs are denoted as *X* = {*x*_*t*_} and *Y* = {*y*_*t*_}. *X* and *Y* are converted to ordinal time series. The marginal probability distribution functions of *X* and *Y* are denoted as *p*(*π*_*i*_) and *p*(*π*_*j*_), respectively. The joint probability functions of *X* and *Y* is denoted as *p*(*π*_*i*_, *π*_*j*_). The conditional probability function of *X* given *Y* is denoted as *p*(*π*_*i*_ | *π*_*j*_).

The joint permutation entropy between *X* and *Y* is defined as

(4) $$\begin{array}{c} H\left(X,Y\right)=-\overset{N}{\underset{i=1}{\sum }}\overset{N}{\underset{j=1}{\sum }}p\left(\pi_{i},\pi_{j}\right)\log p\left(\pi_{i},\pi_{j}\right). \end{array}$$

Then, the conditional permutation entropy of *X* given *Y* is defined as

(5) $$\begin{array}{c} H\left(X\;|\;Y\right)=-\overset{N}{\underset{i=1}{\sum }}\overset{N}{\underset{j=1}{\sum }}p\left(\pi_{i},\pi_{j}\right)\log p\left(\pi_{i}\;|\;\pi_{j}\right). \end{array}$$

The PCMI is calculated by the following equations [41]:

(6) $$\begin{array}{c} I_{X\to Y}^{\delta }=H\left(X\;|\;Y\right)+H\left(Y_{\delta }\;|\;Y\right)-H\left(X,Y_{\delta }\;|\;Y\right),\\ I_{Y\to X}^{\delta }=H\left(Y\;|\;X\right)+H\left(X_{\delta }\;|\;X\right)-H\left(Y,X_{\delta }\;|\;X\right), \end{array}$$

where *X*_*δ*_ or *Y*_*δ*_ is an observation derived from the state of the process *X* or *Y* *δ* steps in the future. The information that is transferred from the process *X* (or *Y*) to the process *Y* (or *X*) is defined as

(7) $$\begin{array}{c} I_{X\to Y}=\frac{\sum_{\delta =\delta_{1}}^{\delta_{2}}I_{X\to Y}^{\delta }}{\delta_{2}-\delta_{1}+1},\\ I_{Y\to X}=\frac{\sum_{\delta =\delta_{1}}^{\delta_{2}}I_{Y\to X}^{\delta }}{\delta_{2}-\delta_{1}+1}, \end{array}$$

where *δ*_1_ and *δ*_2_ are the minimal and maximal steps, respectively. To decrease fluctuations of the estimated directionality index, *I*_*X*→*Y*_ and *I*_*Y*→*X*_ were averaged over a short range of steps. Based on the conditional mutual information, the directionality index between *X* and *Y* is defined by

(8) $$\begin{array}{c} D_{XY}=\left(\frac{I_{X\to Y}-I_{Y\to X}}{I_{X\to Y}+I_{Y\to X}}\right). \end{array}$$

The value of *D*_*XY*_ ranges from −1 to 1. *D*_*XY*_ > 0 means that the information flows from the process *X* to *Y*. *D*_*XY*_ < 0 means that the information flows from the process *Y* to *X*, and *D*_*XY*_ = 0 means that the interactions between *X* and *Y* are symmetrical [41].

Here the length *m* of the patterns was set to 3. To reliably estimate these relative frequencies of each pattern, the length of moving window was set to 2,500 sample points corresponding to 5 s duration and shifted forward in 1-s steps. The lag *τ* = 1. *δ*_1_ and *δ*_2_ were set to 1 and 10, respectively.

### 2.8. Thalamic Inactivation

In order to confirm the results of the data analysis above, we performed the pharmacological experiments. We used the sodium channel blocker tetrodotoxin (TTX) to inactivate the thalamus and examined its effect on the seizure development. These experiments were performed on anesthetic mice because of the limitation of our present experiment conditions. Mice were anesthetized by pentobarbital sodium (100 mg/kg) intraperitoneally and mounted in a stereotaxic frame. Prior to injection of TTX, animals had a baseline seizure induced by the intraperitoneal injection of pilocarpine (300 mg/kg). To deliver drugs to the thalamus, a 1-*μ*L syringe was loaded with either 0.1 mM TTX or 0.9% saline solution for control. The tip of the syringe was placed into PF (AP, −2.3 mm; ML, −0.5 mm; DV, −3.0 mm). The standard TTX injection was 0.8 *μ*L (0.1 mM), released slowly over several minutes. LFPs from CA1 (AP, −2.3 mm; ML, −2.1 mm; DV, −1.4 mm) and PF (AP, −2.3 mm; ML, −0.5 mm; DV, −3.4 mm) were simultaneously recorded with the drug delivery. Electrodes or syringe needles outside the target regions were excluded in the analysis.

## 3. Results

**Figure 2:**
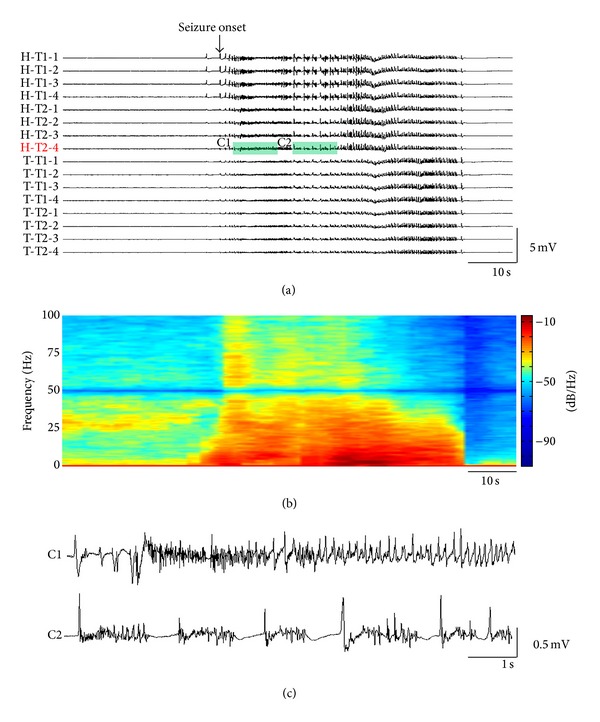
LFPs during the first seizure of an example mouse. (a) Single seizure recorded by four tetrodes. All data were filtered at 0.5–100 Hz. The seizure onset time is marked by an arrow. (b) Power spectral of LFP from Channel H-T2-4. (c) The details of the recordings shown in the shadow in (a). (C1) shows the tonic phase of epileptic seizure and (C2) shows the clonic phase of epileptic seizure. H, hippocampus; T, thalamus; T1&T2, Tetrode 1 and Tetrode 2 in a bundle.

Neural electrical activities were simultaneously recorded from CA1 of hippocampus and PF of thalamus during the development of seizures from 9 mice. Typically, seizures initiated 12–65 min after pilocarpine was injected (31.7  ±  17.8 min, mean  ±  S.D., *n* = 9) and manifested both behaviorally and electrographically. The seizure onset time of seizures was determined by visual inspection based on LFP recordings and confirmed by at least three authors. Clear seizure activities with high-voltage discharges were firstly identified, which usually exceeded the threshold of three times the standard deviation of the baseline. Meanwhile the behavioral severity should be over Stage 4 based on Racine's scale [27]. Then we looked back on the signals with the clear seizure activity to find out the earliest appearance of ictal discharges by eye. In this study, seizures usually started with the hypersynchronous-onset type. Occasionally, seizures started with a period of low-amplitude, high-frequency oscillations, which would develop into high-amplitude, low-frequency oscillations in a few seconds. These two kinds of seizure onset types were very similar as described previously [42]. Generally, the seizures started almost simultaneously from the hippocampus and the thalamus (Figure 2(a)), and the power of LFPs raised immediately over a wide frequency band, including theta, alpha, beta, and gamma activity (Figure 2(b)). Because we used a 50 Hz notch filter when collecting the data, the power density spectral showed a low-power frequency band around 50 Hz. The seizures lasted 20 s up to 73 s (41.3 ± 21.3 s, mean ± S.D., *n* = 9) and then were terminated. Figure 2(c) shows the tonic phase and clonic phase of the seizure shown in Figure 2(a). After the first seizure, recurrent seizures were followed with intervals of 10 to 30 min. In order to obtain comparable results, only the first seizure in each mouse was included in the analysis below.

### 3.1. Redundancy of LFP and Cross-Correlation of Single-Unit Activities

Redundancy of LFP reflects the irregularity of LFP signals. More “monomorphic” LFP yields higher redundancy, while more “pleomorphic” LFP yields lower redundancy. The cross-correlation of single-unit activities reflects the synchronization of firing between neurons. We used these two measures to qualify the dynamic changes of neuronal activities in hippocampus or thalamus during the development of epileptic seizures in different spatial scales.

**Figure 3:**
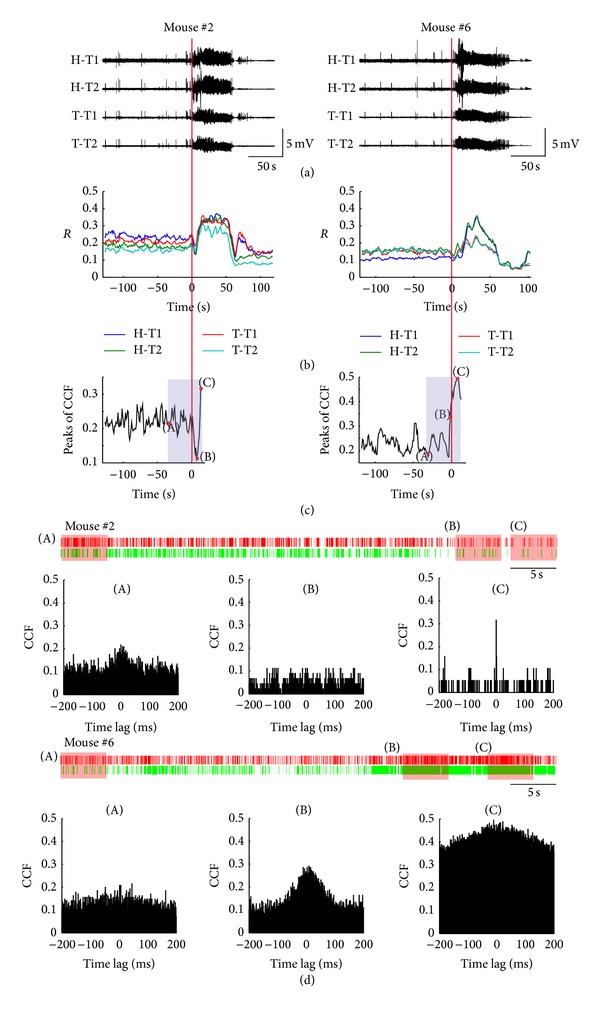
Peri-ictal changes of electrical brain activities as assessed by redundancy (*R*) of LFP and cross-correlation function (CCF) of single-unit activities. (a) LFPs of two sample seizures from Mouse #2 and Mouse #6. Seizure onset is marked by red line. (b) Peri-ictal changes of *R* of LFPs during the seizures shown in (a). (c) Dynamic changing of peak values of CCF of two neuronal spike trains sorted from tetrode H-T2 in Mouse #2 and tetrode T-T1 in Mouse #6. (d) Raster of the spikes from two neurons sorted from the two tetrodes above (shadows in (c)) and CCF in the periods of 5 s marked by (A), (B), and (C) in (c). H, hippocampus; T, thalamus; T1&T2, Tetrode 1 and Tetrode 2 in a bundle.

The redundancy (*R*) of LFP for each tetrode was computed. The results for two example mice (Mouse #2 and #6) are displayed in Figure 3. *R* increased shortly after the seizure onset and then followed by a fluctuating time course with an overall tendency to decrease toward the seizure termination and into the post-seizure time period (Figure 3(b)). *R* maintained at high level throughout the seizure for Mouse #2, while *R* reached its maximum value at the beginning half of seizure and then slowly decreased to the end for Mouse #6. The trends of *R* corresponding to the evolution of the two seizures were consistent, though the details were different.

**Figure 4:**
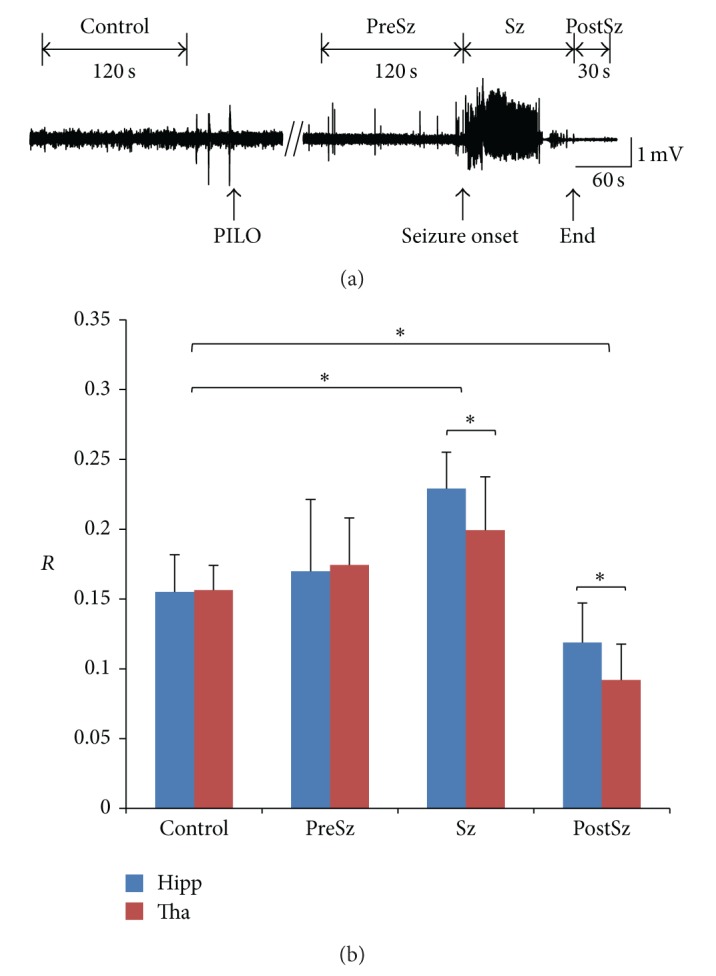
Statistics analysis result of signal redundancy for all 9 mice. (a) Time periods' definition. Control period contains 120 s before the pilocarpine injection. Pre-seizure (PreSz) time period is before the seizure onset with 120-s long. Post-seizure (PostSz) time period is immediately after seizure termination with 30 s long. Time period between seizure onset and termination is denoted as “Sz.” (b) Statistical analysis results of redundancy (*R*). Error bar means standard deviation. Stars (∗) indicate statistically significant differences between the distributions for control period and the following three time periods (pair-wised *t*-tests, significance level *P* < 0.05). Hipp, hippocampus; Tha, thalamus; PILO, pilocarpine.

The statistics analysis results across all of the 9 seizures from 9 mice are illustrated in Figure 4. The pre-seizure time period (PreSz) contains 120 s before the seizure onset, and the post-seizure time period (PostSz) contains 30 s immediately after the seizure termination. Here we chose the time course of PostSz shorter than that of PreSz. Because the mouse would soon come out of the depressed pos-tseizure state and began preparing for the next seizure about 30 s after the seizure termination based on our experiences. The time period between the seizure onset and termination is denoted as “Sz,” whose period varies from seizure to seizure, lasting 20 s to 73 s. The control period contains 120 s before the pilocarpine injection (Figure 4(a)). As shown in Figure 4(b), in both hippocampus and thalamus, *R* of Sz was significantly larger than control (pair-wised *t*-test, *P* < 0.05, *n* = 9), and *R* of PostSz was significantly smaller than control (pair-wised *t*-test, *P* < 0.05, *n* = 9). There was no significant difference between control and PreSz period (Figure 4(b)). This indicated that the LFPs during the seizure are more “monomorphic” and rhythmic. In addition, *R* of Sz and PostSz in hippocampus were significantly larger than that in thalamus, but there was no significant difference during the control and PreSz periods. This indicated that during the Sz and PostSz periods LFPs in hippocampus were more rhythmic than those in thalamus.

In some cases, *R* showed a transient decrease at the seizure onset, which may last 6 to 12 s (9.1 ± 2.3 s, mean ± S.D., *n* = 4) (Figure 3(b)). 4 of 9 seizures showed this phenomenon. It reflected that the patterns of LFPs become more random at the seizure onset, but the random state would not last long and it would be soon replaced by the rhythmic state.

We further analyzed the single-unit activities recorded from tetrode H-T2 in Mouse #2 and tetrode T-T1 in Mouse #6. Two neurons were sorted from each tetrode (Figure 3(d)). It is difficult to reliably identify the single units during the seizure when the LFP showed sharp ictal discharges. So only the first few seconds after the seizure onset were included in the analysis. We computed the CCF of spike trains. It showed a transient desynchronization of neuronal activities at the seizure onset and a resynchronization phase in the flowing period in Mouse #2, while it did not show the desynchronization phase in Mouse #6 (Figures 3(c) and 3(d)).

As shown in Figures 3(b) and 3(c), the variation tendency of the peak values of CCF between neurons matched the variation tendency of *R* of LFP. As we know, LFP is a summation of synaptic activities of many of neurons around the recording electrode. It reminds us that the rhythmic LFP might reflect the synchronization of neurons to some extent. In some way, the dynamic of LFP could reflect the interactions between neurons, including the synchrony characteristics. Unfortunately, we could not get the statistical analysis results of the cross-correlation of single neurons, since we could only get the recordings from a few of single neurons steadily during the induction of seizures at the present experiment conditions.

### 3.2. Directionality Index between Hippocampus and Thalamus

**Figure 5:**
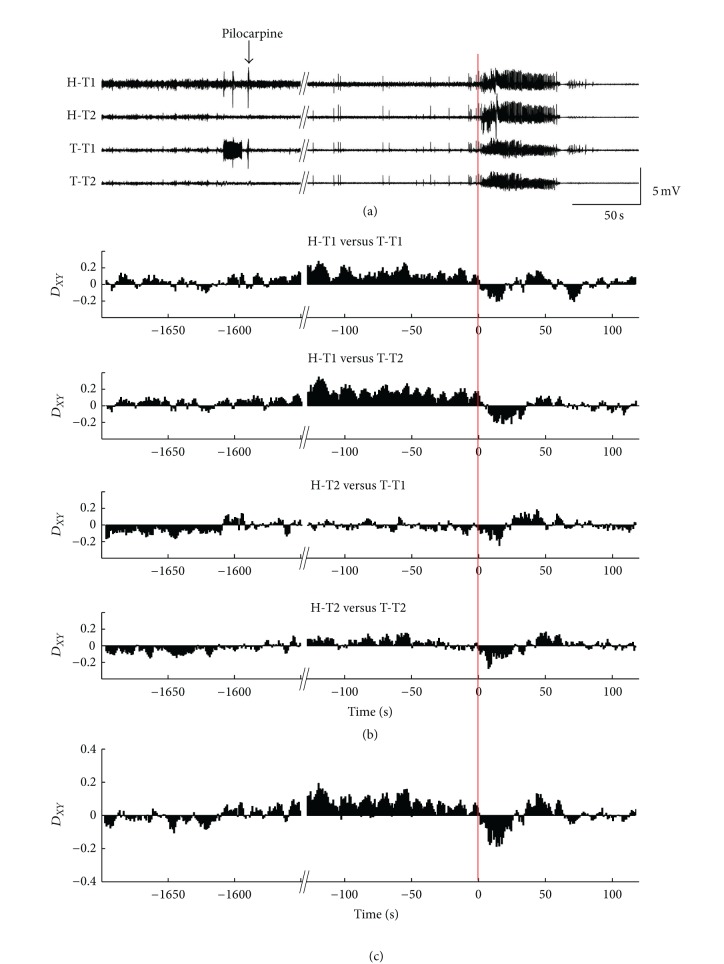
One example of peri-ictal coupling dynamics between hippocampus and thalamus. (a) Mean values of LFPs recorded from four channels of each tetrode. (b) The directionality index (*D*_*XY*_) between hippocampus and thalamus. We calculated *D*_*XY*_ between each tetrode's recording from hippocampus and thalamus of the example mouse, getting four combinations. (c) The averaged directionality index (*D*_*XY*_). The red line indicates the seizure onset time. H, hippocampus; T, thalamus; T1&T2, Tetrode 1 and Tetrode 2 in a bundle.

To assess the interaction between two neuronal populations recorded from hippocampus and thalamus, we computed directionality index (*D*_*XY*_) between LFP signals of the two areas.  Figure 5(a) shows the LFPs recorded by four tetrodes during the first seizure from Mouse #2. The red line indicates the seizure onset time. *D*_*XY*_ between LFP signals of the two areas are displayed in Figure 5(b) and the averaged *D*_*XY*_ is displayed in Figure 5(c). The results showed that the information flowed from hippocampus to thalamus before seizure, and inversely, the information flowed from thalamus to hippocampus at the seizure onset. After about 30 s, the direction of information flows recovered. After the seizure termination, *D*_*XY*_ was very small, which means the interaction between hippocampus and thalamus were almost symmetrical.

**Figure 6:**
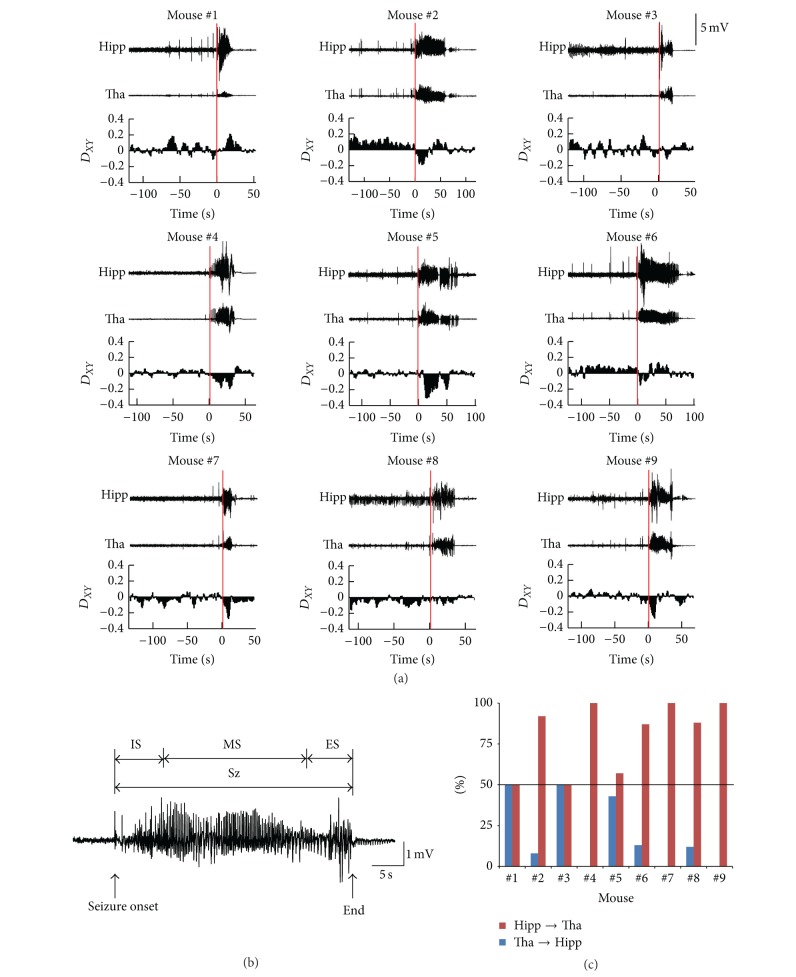
Peri-ictal coupling dynamics between hippocampus and thalamus. (a) Time course of directionality index (*D*_*XY*_) between hippocampus and thalamus during the epileptic seizures in 9 mice. The upper two traces show the averaged LFPs from hippocampus and thalamus. The lower trace shows the averaged *D*_*XY*_. The red line indicates the seizure onset time. (b) Time periods' definition. We divided the Sz period into three parts. The first part is the initiation period of seizure (IS), containing one-fifth of the time course of seizure from the seizure onset. The third part is the end of seizure (ES), containing one-fifth of the time course of seizure immediately before the termination of seizure. The second part is the middle part of the seizure (MS) between the first and the third part. (c) Percentages of coupling direction “hippocampus → thalamus” and “thalamus → hippocampus” during the initiation period of seizure (IS). The black solid line marks the threshold of 50%. Hipp, hippocampus; Tha, thalamus.

We inspected *D*_*XY*_ between hippocampus and thalamus of all seizures (Figure 6(a)). The results show that the information flowed from thalamus to hippocampus at the seizure onset or shortly after the seizure onset in most cases, no matter what the direction was before the seizure onset or after the early part of the seizure. For the statistics analysis, we further divided the Sz period into three parts. The first part is the initiation period of the seizure (IS), containing one-fifth of the time course of the seizure from the seizure onset. The third part is the end of the seizure (ES), containing one-fifth of the time course of the seizure immediately before the seizure termination. The second part is the middle part of the seizure (MS) between the first and the third part (Figure 6(b)). We then calculated the percentages of the two directions of information flow during the three periods of the seizure for each mouse. We obtained *D*_*XY*_ in each moving window during the three periods. For a certain period and for each mouse, we defined the percentage of *D*_*XY*_ > 0 as the percentage of information flow from hippocampus to thalamus and the percentage of *D*_*XY*_ < 0 as the percentage of information flow from thalamus to hippocampus, respectively. For each mouse, the summation of percentages of the two directions is 100% during each specific period. For the IS period, the information flowed from thalamus to hippocampus in most cases (7 of 9 seizures, including Mouse #2, #4, #5, #6, #7, #8, and #9). In the other two seizures, the information flow was symmetrically bidirectional, but none was found in which the information flowed from hippocampus to thalamus during the IS period (Figure 6(c)). In the other two periods, we did not find consistent results (data not shown). These results suggest that the thalamus may play an important role in the initiation of seizure.

### 3.3. Inactivation of Thalamus

**Figure 7:**
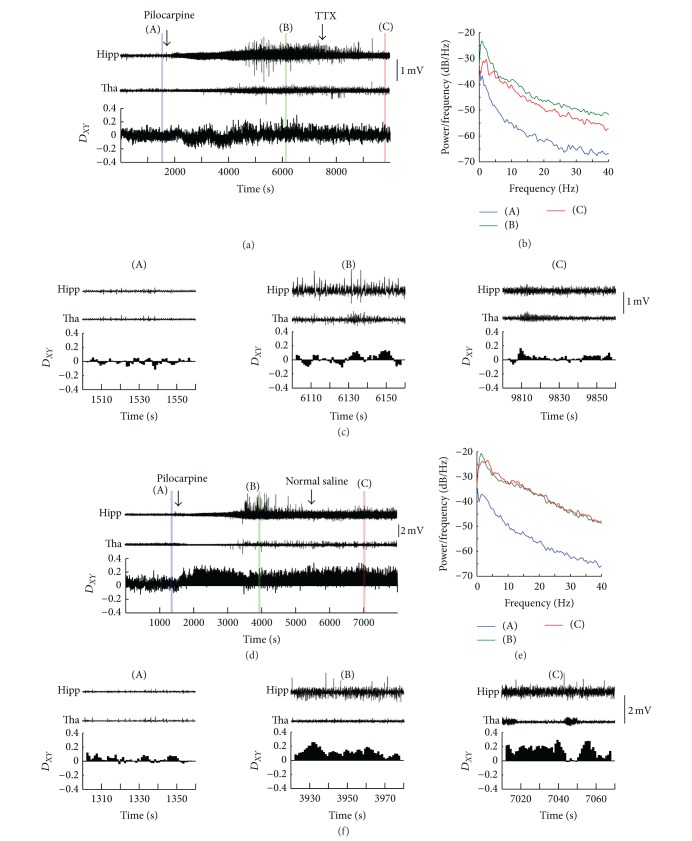
Seizures affected by the TTX injection in thalamus. (a) LFPs from hippocampus and thalamus and the averaged directionality index (*D*_*XY*_) between the two brain areas before and after the injection of TTX into thalamus. (b) The power spectral density of the LFPs in hippocampus (shadowed areas in (a)). (c) 60-second samples from each trace in (a) (the shadowed areas). (d) LFPs from hippocampus and thalamus and averaged directionality index (*D*_*XY*_) between the two brain areas before and after the injection of normal saline into thalamus in a control mouse. (e) The power spectral density of the LFPs in hippocampus (shadowed areas in (d)). (f) 60-second samples from each trace in (d) (the shadowed areas). Hipp, hippocampus; Tha, thalamus.

To further confirm the role of thalamus, we used the pharmacological method to inactivate thalamus to examine its effect on the seizure. After the animals had a baseline seizure induced by pilocarpine, TTX was injected into the thalamus (*n* = 2). The inactivation of thalamus caused a reduction of seizure activities (Figures 7(a) and 7(c)), and there was a power reduction as shown in Figure 7(b), though the power was larger than the baseline. But the injections of normal saline (*n* = 1) had little effect on the development of seizure activities and the power spectral (Figures 7(d)–7(f)). In both cases, the directionality index (*D*_*XY*_) did not show obvious change after the injection of TTX or normal saline (Figures 7(a) and 7(d)). These results indicated that PF did not play the leading role after seizure initiation, but it still had an effect on the seizure activities.

## 4. Discussion

In this study, we used 16-channel microelectrode to record the LFPs and single-unit activities from hippocampus and thalamus of mice. The computational methods from symbolic dynamics and information theory were used to investigate the dynamic changes of neural activities from each brain area and the direction of information flow between the two brain areas. These methods mapped the raw LFP signals into ordinal time series, which reflected the relational aspects between consecutive values of the original LFP signals but not the values themselves. Our findings, mainly based on analysis of LFPs, reveal two important points about the epileptic seizures induced by pilocarpine.

First, we found a global increase of redundancy of ictal LFPs, either in hippocampus or in thalamus (Figures 3 and 4). However, in some cases, the redundancy of LFP (*R*) shows a transient decrease shortly after the seizure onset, which may last 6 to 12 s (9.1 ± 2.3 s, mean ± S.D., *n* = 4) (Figure 3(b)). The increase of *R* suggests more rhythmic LFPs occurred during the ictal state, while the transient decrease of *R* suggests that the LFPs are transiently disordered at the seizure onset. In a traditional view, epileptic seizures are commonly considered to be the result of monolithic, hypersynchronous activities arising from an imbalance between the excitation and inhibition in neuronal network. However, our results indicate that the epileptic seizure is not a monolithic state. In recent years, several studies have demonstrated the heterogeneity of epileptic seizure in different spatial scales. Schindler and his colleagues find that the correlation of multichannel EEG either remains approximately unchanged or decreases during the first half of the seizures in patients with pharmacoresistant focal epilepsy [43]. In a smaller spatial scale, studies indicate that the LFP synchrony between the seizure generating brain and the other brain regions is lower in epilepsy patients than in control patients [44]. Another study shows that fractured microdomains exist in the epileptic brain, which can be observed by isolated microelectrodes rather than clinical macroelectrodes [45]. In a much smaller scale, it was shown that neuronal spiking activity during the initiation and spreading of seizures in humans was highly heterogeneous, suggesting complex and variable interactions between different neuronal groups [46].

Since LFPs are a summation of the synaptic activities of many of neurons around the recording electrode, we then examined how the single neurons acted during the peri-ictal period. The variation tendency of the peak values of CCF between neurons matches the variation tendency of *R* of LFP recorded from the same electrode very well, even in the period of transient decrease of *R*. As shown in Figures 3(b) and 3(c), when the CCF peak value gets larger, *R* gets larger as well. CCF of single neurons pair is a measure of the synchronization of neuronal activities. So we speculate that the lager *R* may reflect higher level of the synchronization of single neurons to some extent. The global increase of *R* during the seizures may reflect the global synchronization of neurons around the electrode to some extent, while the transient decrease of *R* may reflect the transient desynchronization of neurons around the electrode to some extent. Interestingly, Cymerblit-Sabba and Schiller find the similar biphasic network dynamics, which composed of an early desynchronization phase and a late resynchronization phase, in the pre-ictal state of pharmacologically induced seizures in rats [47]. The biphasic network dynamics may reveal a specific network mechanism underlying the development of epileptic seizure. But unfortunately, we do not have enough evidence to confirm this now. We cannot record lager amount of single-unit activities during the development of seizure in the present experiment conditions. We will try to improve the recording techniques to collect much more single-unit firing activities to confirm our conclusion.

Second, we found that the information tended to flow from thalamus to hippocampus during the initiation period of seizure (Figures 5 and 6). This indicates that the thalamus led the hippocampus during the initiation period to some extent. In clinical study, it shows the similar coupling direction between hippocampus and thalamus during the seizure initiation period in some of patients with TLE [9]. In animal models of TLE, studies have shown that thalamus indeed plays an important role in the initiation and spread of seizures, though the exact role is less understood [18, 19, 21–25]. Bertram put forward a framework to explain the neuronal circuits that support the different stages of seizures. It contains four parts, seizure focus, initiating circuits, paths of spread and neuromodulatory centers. In this framework, thalamus is served as part of the initiating circuit, which is the separate neuronal populations that are necessary to support the start of a seizure [10]. Although the framework need to be confirmed using a larger amount of data in the future, it provides a new view point to consider the role of brain structures out of the temporal lobe in TLE at the level of neuronal circuits. Our results are consistent with this framework, suggesting thalamus may play an important role in the initiation of epileptic seizures induced by pilocarpine. On the contrary, studies by Toyoda and colleagues have demonstrated that dorsomedial thalamus has consistently late seizure onsets and is unlikely involved in the seizure initiation in pilocarpine-treated rat model [48]. The controversial conclusion may be due to the different recording sites or different animal species. In addition, they drew the conclusion by simply comparing the seizure onset time across different brain regions. Without detectable epileptiform discharges in thalamus at the first moment of seizure onset does not mean that there is no information exchange between thalamus and the brain areas where the seizure starts, and thalamus may be involved in the seizure initiation circuits. Applying computational methods, such as PCMI, to analysis LFPs may reveal the characteristics of epileptic network which might not be accessible by the examination of the waveforms of neuronal signals. Of course, we cannot draw a strong conclusion only by the mathematical results. But we provide a new perspective to understand the seizure generation induced by pilocarpine in mice.

The pharmacological experiments show a suppression effect on the seizure activities by injection of TTX into thalamus but not completely. The power of LFPs in hippocampus after TTX injection is smaller than that before TTX injection but is higher than the baseline (Figure 7). Since the injection site was located at PF of thalamus, this result reminds us that the PF of thalamus may be not the only structure which has effect on the development of seizure, but it does have effect partially.

The TTX delivery experiment was done at the anesthetic state because of the limitation of our experiment conditions. TTX was injected into PF after the mouse had a baseline seizure. It is difficult to induce epileptic seizure by pilocarpine under anesthetic state. If TTX was injected before the seizure induction, we cannot determine whether the TTX injection had effect on the seizure or the seizure was not induced at all. So we cannot observe the effect of TTX on the initiation of epileptic seizures. In our experiments, *D*_*XY*_ did not change after TTX injection, suggesting that PF did not play the leading role after seizure initiation. This result does not conflict with our results in freely moving state, nor can confirm the results before. So we will keep working on it in freely moving state in the future.

From a therapeutic perspective, researches on the role of thalamus in TLE may provide a potential target to the clinical resection and the deep brain stimulation. They may also further the understanding of the nature of seizure initiation and spread. Our data analysis brought new insight into the dynamic changes of single brain area and the interaction between brain areas during the development of seizures, and we still have many jobs to improve and further confirm our results.
